# Supplementary material for: Impact of E484Q and L452R Mutations on Structure and Binding Behavior of SARS-CoV-2 B.1.617.1 Using Deep Learning AlphaFold2, Molecular Docking and Dynamics Simulation
Source: Int J Mol Sci. 2023 Jul 17;24(14):11564. doi: 10.3390/ijms241411564 (PMC10380202; doi:10.3390/ijms241411564)
Supplement: Supplementary file 1 [file ijms-24-11564-s001.zip › ijms-2464713-supplementary.pdf]

Supplementary Materials

Impact of E484Q and L452R Mutations on Structure and Binding Behavior of SARS-CoV-2 B.1.617.1 Using Deep Learning AlphaFold2, Molecular Docking and Dynamics Simulation

Yanqi Jiao, Yichen Xing and Yao Sun *

School of Science, Harbin Institute of Technology (Shenzhen), Shenzhen 518055, China; abigcat1204@gmail.com (Y.J.); 22s058083@stu.hit.edu.cn (Y.X.)
* Correspondence: sunyao0819@hit.edu.cn

Table S1. The pLDDT values of the full-length S protein, S1 RBD and S1 NTD of the double mutant variant.

	S Protein	S1 RBD	S1 NTD
**RANK 0**	78.46	85.13	88.10
**RANK 1**	78.98	86.69	88.66
**RANK 2**	65.87	76.50	84.78
**RANK 3**	60.84	73.33	85.42
**RANK 4**	65.91	73.47	86.09
**Mean**	70.01	79.02	86.61

Table S2. The TM, MaxSub, and (GDT)-TS scores of the predicted structures of S proteins of the WT and experimental 7DF4.

	RANK 0	RANK 1
**TM score**	0.814	0.8252
**MaxSub score**	0.6202	0.6836
**(GDT)-TS score**	0.6041	0.6598
**RMSD**	2.477	1.95

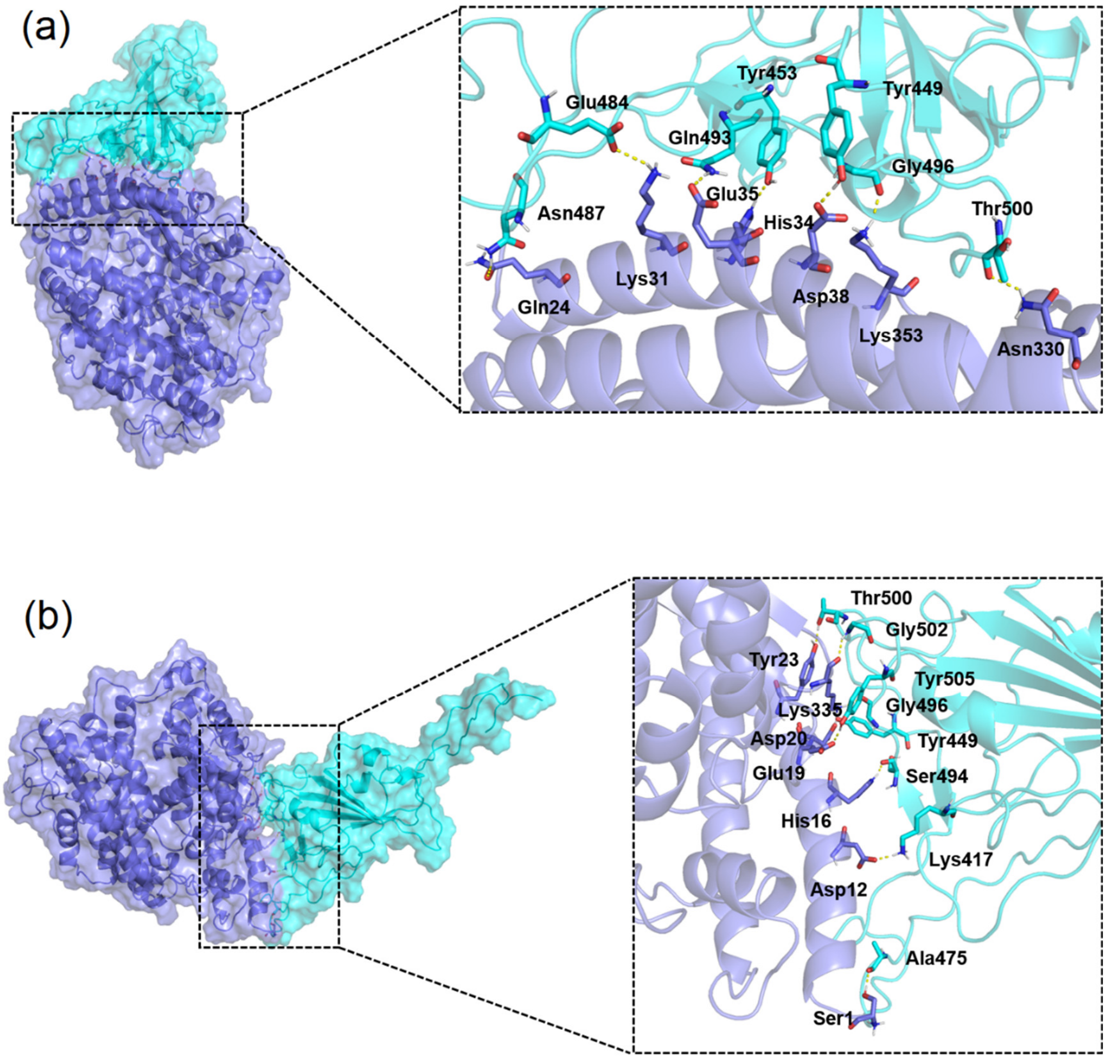

Figure S1. (**a**) Interface residues between the docked S1 RBD of WT and hACE2. (**b**) Interface residues between the docked S1 RBD of the double mutant variant and hACE2. S1 RBDs are shown in cyan, while hACE2 is shown in purple. Interface residues of S1 RBDs and hACE2 are highlighted in the boxes at right.
